# Poly[(μ_3_-quinoline-6-carboxyl­ato-κ^3^ *N*:*O*:*O*′)silver(I)]

**DOI:** 10.1107/S1600536812023835

**Published:** 2012-05-31

**Authors:** Chun-Wei Yeh, Ay Jong, Chi-Hui Tsou, Fu-Chang Huang, Maw-Cherng Suen

**Affiliations:** a Department of Chemistry, Chung-Yuan Christian University, Jhongli 32023, Taiwan; b Department of Applied Cosmetology, Taoyuan Innovation Institute of Technology, Jhongli 32091, Taiwan; c Department of Materials Science and Engineering, National Taiwan University of Science and Technology, Taipei 10607, Taiwan; d Department of Civil and Environmental Engineering, Department of Materials Science and Engineering, Taoyuan Innovation Institute of Technology, Jhongli 32091, Taiwan; e Department of Materials and Fibers, Taoyuan Innovation Institute of Technology, Jhongli 32091, Taiwan

## Abstract

In the title coordination polymer, [Ag(C_10_H_6_NO_2_)]_*n*_, the Ag^I^ cation is coordinated by two O atoms and one N atom from three 6-quinoline­carboxyl­ate anions in a distorted T-shaped AgNO_2_ geometry, in which the O—Ag—O angle is 160.44 (9)°. The 6-quinoline­carboxyl­ate anion bridges three Ag^+^ cations, forming a nearly planar polymeric sheet parallel to (101). The distance between Ag^+^ cations bridged by the carboxyl group is 2.9200 (5) Å. In the crystal, π–π stacking is observed between parallel quinoline ring systems, the centroid–centroid distance being 3.7735 (16) Å.

## Related literature

For background to coordination polymers with organic ligands, see: Kitagawa *et al.* (2004 ▶); Chiang *et al.* (2008 ▶); Yeh *et al.* (2008 ▶, 2009 ▶); Hsu *et al.* (2009 ▶). For related pyridine­carboxyl­ate structures, see: Yeh *et al.* (2004 ▶); Ockwig *et al.* (2005 ▶); Chen *et al.* (2008 ▶); Hirano *et al.* (2002 ▶) and for related 6-quinoline­carboxyl­ate structures, see: Lin & Maggard (2007 ▶); Du *et al.* (2008**a* ▶,b* ▶); Hu *et al.* (2008 ▶); Xu *et al.* (2009 ▶).
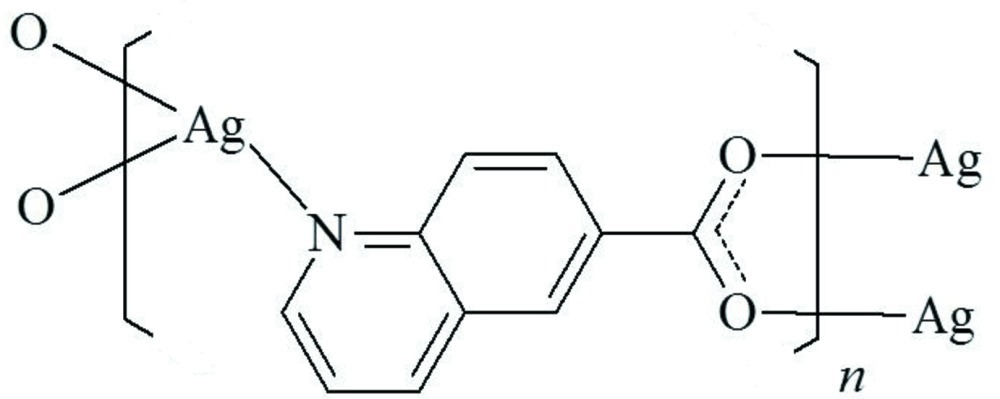


## Experimental

### 

#### Crystal data

[Ag(C_10_H_6_NO_2_)]
*M* *_r_* = 280.03
Monoclinic, 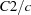

*a* = 13.0008 (10) Å
*b* = 14.3900 (11) Å
*c* = 9.3431 (7) Å
β = 103.446 (1)°
*V* = 1700.0 (2) Å^3^
*Z* = 8
Mo *K*α radiation
μ = 2.34 mm^−1^
*T* = 294 K
0.39 × 0.28 × 0.25 mm

#### Data collection

Bruker APEXII CCD diffractometer
Absorption correction: multi-scan (*SADABS*; Bruker, 2000 ▶) *T*_min_ = 0.471, *T*_max_ = 1.000
4717 measured reflections
1674 independent reflections
1543 reflections with *I* > 2σ(*I*)
*R*_int_ = 0.021

#### Refinement

*R*[*F* ^2^ > 2σ(*F* ^2^)] = 0.027
*wR*(*F* ^2^) = 0.075
*S* = 1.08
1674 reflections
127 parameters
H-atom parameters constrained
Δρ_max_ = 0.37 e Å^−3^
Δρ_min_ = −0.94 e Å^−3^

### 

Data collection: *APEX2* (Bruker, 2010 ▶); cell refinement: *SAINT* (Bruker, 2010 ▶); data reduction: *SAINT*; program(s) used to solve structure: *SHELXS97* (Sheldrick, 2008 ▶); program(s) used to refine structure: *SHELXL97* (Sheldrick, 2008 ▶); molecular graphics: *DIAMOND* (Brandenburg, 2010 ▶); software used to prepare material for publication: *SHELXL97*.

## Supplementary Material

Crystal structure: contains datablock(s) I, New_Global_Publ_Block. DOI: 10.1107/S1600536812023835/xu5546sup1.cif

Structure factors: contains datablock(s) I. DOI: 10.1107/S1600536812023835/xu5546Isup2.hkl

Additional supplementary materials:  crystallographic information; 3D view; checkCIF report

## Figures and Tables

**Table 1:** Selected bond lengths (Å)

Ag—O1^i^	2.2067 (19)
Ag—O2^ii^	2.252 (2)
Ag—N	2.397 (2)

Symmetry codes: (i) 
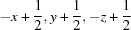
; (ii) 
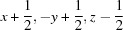
.

**Fig. 1.**
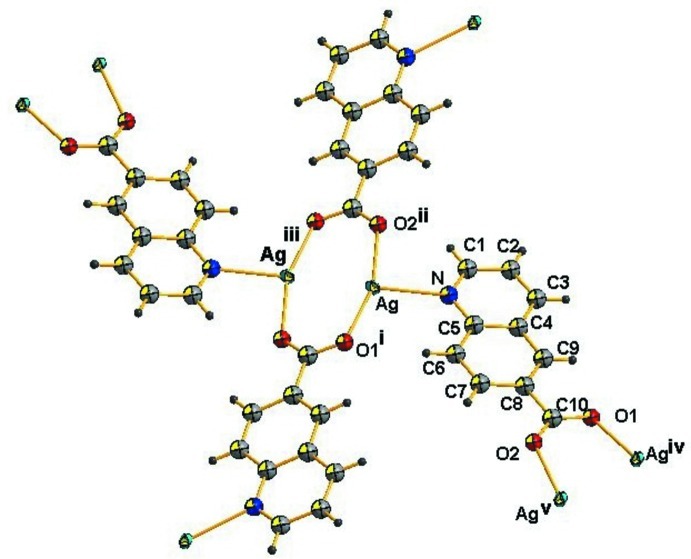
A portion of the two-dimensional grid. Ellipsoids are drawn at 30% probability level. Symmetry codes: (i) -x + 1/2,y + 1/2,-z + 1/2;(ii) x + 1/2,-y + 1/2,z - 1/2; (iii) -x + 1,-y + 1,-z; (iv) -x + 1/2,y - 1/2,-z + 1/2; (v) x - 1/2,-y + 1/2,z + 1/2.

**Fig. 2.**
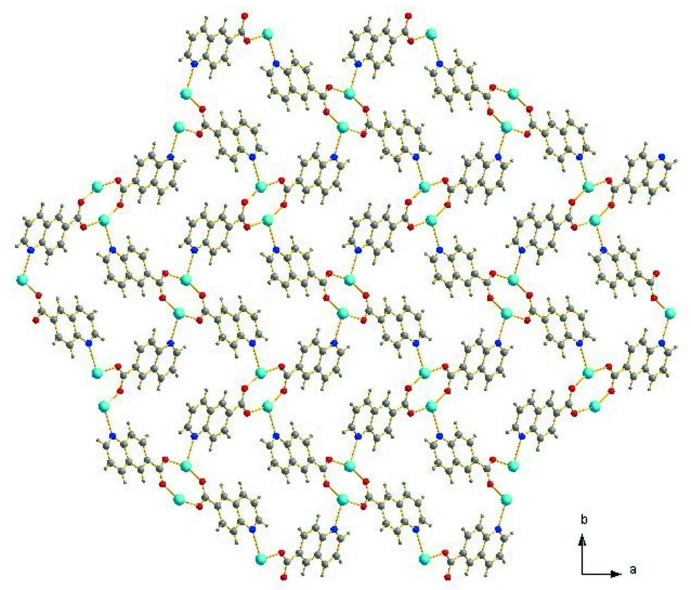
The view shows the pleated (4,4) net along (001) direction.

**Fig. 3.**
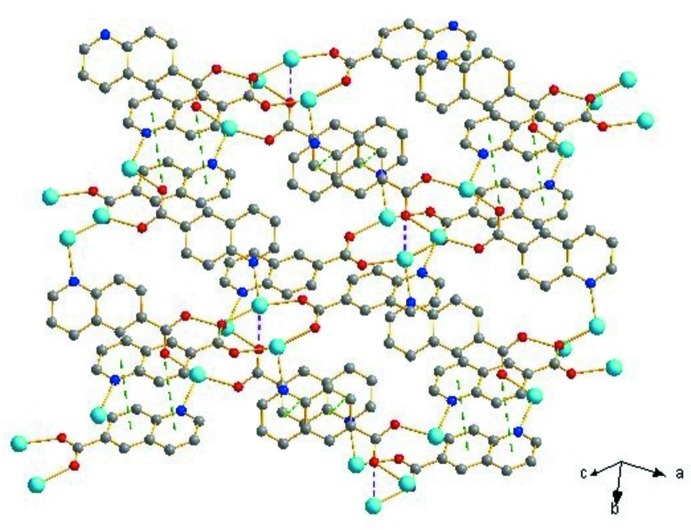
The packing diagram shows the Ag···O interactions and π-π stacking interactions between the two-dimensional networks.

## supplementary crystallographic information

### Comment

The synthesis of metal coordination polymers has been a subject of intense research due to their interesting structural chemistry and potential applications in gas storage, separation, catalysis, magnetism, luminescence, and drug delivery (Kitagawa *et al.*, 2004). Roles of anion, solvent and ligand comformations in self-assembly of coordination complexes containing polydentate nitrogen ligands are very intersting (Chiang *et al.*, 2008; Yeh *et al.*, 2008; Hsu *et al.*, 2009; Yeh *et al.*, 2009). In the past, the pyridinecarboxylate ligands have been subjected to many studies of its coordination ability to metal centers (Yeh *et al.*, 2004; Ockwig *et al.*, 2005; Chen *et al.*, 2008; Hirano *et al.*, 2002). The various metal complexes containing 6-quinolinecarboxylate (*L^-^*) ligands have been reported, which show various multi-dimensional frameworks (Lin & Maggard, 2007; Du *et al.*, 2008**a*,b*; Hu *et al.*, 2008; Xu *et al.*, 2009). The Ag^+^ cations are coordinated with two N atoms from two 1,2-bis(4,4-dimethyl-4,5-dihydrooxazol-2-yl)ethane (*L*) ligands (Fig. 1). The Ag···Ag distances separated by the bridging *L^-^* anions are 2.9200 (5), 9.974 (1) and 10.469 (1) Å, while the unit of dinuclear Ag^+^ are forming (4,4) polymeric nets (Fig. 2). The two-dimensional polymeric nets are interlinking through Ag···O interactions [2.954 (2) Å] and pi—pi stacking interactions in the crystal structure (Fig. 3).

### Experimental

An aqueous solution (5.0 ml) of AgNO_3_ (1.0 mmol) was layered carefully over a methanolic solution (5.0 ml) of 6-quinolinecarboxylic acid (1.0 mmol) in a tube and kept it in the dark. Colourless crystals were obtained after several weeks.

### Refinement

All the H atoms were constrained to ideal geometries, with C—H = 0.93 Å (aromatic) and *U*_iso_(H) = 1.2*U*_eq_(C).

### Crystal data

**Table:**

[Ag(C_10_H_6_NO_2_)]	*F*(000) = 1088
*M**_r_* = 280.03	*D*_x_ = 2.188 Mg m^−^^3^
Monoclinic, *C*2/*c*	Mo *K*α radiation, λ = 0.71073 Å
Hall symbol: -C 2yc	Cell parameters from 3671 reflections
*a* = 13.0008 (10) Å	θ = 2.2–26.0°
*b* = 14.3900 (11) Å	µ = 2.34 mm^−^^1^
*c* = 9.3431 (7) Å	*T* = 294 K
β = 103.446 (1)°	Block, colourless
*V* = 1700.0 (2) Å^3^	0.39 × 0.28 × 0.25 mm
*Z* = 8	

### Data collection

**Table:**

Bruker APEXII CCD diffractometer	1674 independent reflections
Radiation source: fine-focus sealed tube	1543 reflections with *I* > 2σ(*I*)
Graphite monochromator	*R*_int_ = 0.021
φ and ω scans	θ_max_ = 26.0°, θ_min_ = 2.1°
Absorption correction: multi-scan (*SADABS*; Bruker, 2000)	*h* = −16→7
*T*_min_ = 0.471, *T*_max_ = 1.000	*k* = −17→17
4717 measured reflections	*l* = −11→11

### Refinement

**Table:**

Refinement on *F*^2^	Primary atom site location: structure-invariant direct methods
Least-squares matrix: full	Secondary atom site location: difference Fourier map
*R*[*F*^2^ > 2σ(*F*^2^)] = 0.027	Hydrogen site location: inferred from neighbouring sites
*wR*(*F*^2^) = 0.075	H-atom parameters constrained
*S* = 1.08	*w* = 1/[σ^2^(*F*_o_^2^) + (0.0444*P*)^2^ + 2.6516*P*] where *P* = (*F*_o_^2^ + 2*F*_c_^2^)/3
1674 reflections	(Δ/σ)_max_ = 0.001
127 parameters	Δρ_max_ = 0.37 e Å^−^^3^
0 restraints	Δρ_min_ = −0.94 e Å^−^^3^

### Special details

**Table:**

Geometry. All e.s.d.'s (except the e.s.d. in the dihedral angle between two l.s. planes) are estimated using the full covariance matrix. The cell e.s.d.'s are taken into account individually in the estimation of e.s.d.'s in distances, angles and torsion angles; correlations between e.s.d.'s in cell parameters are only used when they are defined by crystal symmetry. An approximate (isotropic) treatment of cell e.s.d.'s is used for estimating e.s.d.'s involving l.s. planes.
Refinement. Refinement of *F*^2^ against ALL reflections. The weighted *R*-factor *wR* and goodness of fit *S* are based on *F*^2^, conventional *R*-factors *R* are based on *F*, with *F* set to zero for negative *F*^2^. The threshold expression of *F*^2^ > σ(*F*^2^) is used only for calculating *R*-factors(gt) *etc*. and is not relevant to the choice of reflections for refinement. *R*-factors based on *F*^2^ are statistically about twice as large as those based on *F*, and *R*- factors based on ALL data will be even larger.

### Fractional atomic coordinates and isotropic or equivalent isotropic displacement parameters (Å^2^)

**Table:**

	*x*	*y*	*z*	*U*_iso_*/*U*_eq_	
Ag	0.47914 (2)	0.410289 (16)	0.06057 (3)	0.04647 (13)	
O1	0.12021 (17)	−0.00759 (14)	0.3242 (2)	0.0426 (5)	
O2	0.1053 (2)	0.12550 (16)	0.4404 (3)	0.0530 (6)	
N	0.42688 (18)	0.25041 (16)	0.0564 (2)	0.0345 (5)	
C1	0.4658 (2)	0.2003 (2)	−0.0380 (3)	0.0422 (7)	
H1A	0.5138	0.2286	−0.0840	0.051*	
C2	0.4390 (3)	0.1078 (2)	−0.0724 (4)	0.0460 (7)	
H2A	0.4675	0.0765	−0.1412	0.055*	
C3	0.3706 (2)	0.0635 (2)	−0.0040 (3)	0.0398 (6)	
H3A	0.3529	0.0015	−0.0243	0.048*	
C4	0.3274 (2)	0.11373 (19)	0.0982 (3)	0.0303 (5)	
C5	0.3568 (2)	0.20801 (18)	0.1252 (3)	0.0298 (5)	
C6	0.3125 (2)	0.25912 (18)	0.2252 (3)	0.0328 (5)	
H6A	0.3305	0.3213	0.2434	0.039*	
C7	0.2435 (2)	0.21763 (19)	0.2955 (3)	0.0326 (5)	
H7A	0.2150	0.2522	0.3610	0.039*	
C8	0.2145 (2)	0.12338 (19)	0.2705 (3)	0.0297 (5)	
C9	0.2555 (2)	0.07253 (19)	0.1723 (3)	0.0320 (5)	
H9A	0.2360	0.0107	0.1544	0.038*	
C10	0.1398 (2)	0.07663 (19)	0.3510 (3)	0.0324 (6)	

### Atomic displacement parameters (Å^2^)

**Table:**

	*U*^11^	*U*^22^	*U*^33^	*U*^12^	*U*^13^	*U*^23^
Ag	0.0560 (2)	0.03486 (17)	0.0611 (2)	0.00687 (9)	0.03898 (14)	−0.00040 (9)
O1	0.0516 (12)	0.0344 (11)	0.0518 (11)	−0.0060 (9)	0.0324 (10)	−0.0010 (9)
O2	0.0695 (15)	0.0394 (12)	0.0698 (15)	−0.0088 (11)	0.0563 (13)	−0.0094 (11)
N	0.0351 (12)	0.0319 (12)	0.0425 (12)	−0.0010 (9)	0.0209 (10)	0.0046 (9)
C1	0.0440 (16)	0.0419 (16)	0.0501 (15)	−0.0009 (13)	0.0304 (13)	0.0016 (13)
C2	0.0533 (19)	0.0432 (16)	0.0529 (18)	0.0022 (14)	0.0354 (15)	−0.0056 (13)
C3	0.0457 (16)	0.0351 (14)	0.0453 (15)	−0.0009 (13)	0.0246 (13)	−0.0048 (12)
C4	0.0313 (13)	0.0293 (12)	0.0342 (13)	0.0022 (10)	0.0159 (10)	0.0024 (10)
C5	0.0293 (12)	0.0313 (13)	0.0319 (12)	0.0013 (10)	0.0135 (10)	0.0040 (10)
C6	0.0377 (14)	0.0260 (12)	0.0380 (13)	−0.0021 (10)	0.0158 (11)	−0.0021 (10)
C7	0.0359 (13)	0.0328 (14)	0.0334 (12)	0.0022 (11)	0.0168 (10)	−0.0018 (10)
C8	0.0306 (12)	0.0300 (13)	0.0327 (12)	0.0021 (11)	0.0158 (10)	0.0041 (10)
C9	0.0364 (14)	0.0270 (12)	0.0377 (13)	−0.0004 (10)	0.0193 (11)	0.0009 (10)
C10	0.0323 (13)	0.0349 (14)	0.0358 (13)	0.0022 (11)	0.0196 (11)	0.0046 (10)

### Geometric parameters (Å, º)

**Table:**

Ag—O1^i^	2.2067 (19)	C2—H2A	0.9300
Ag—O2^ii^	2.252 (2)	C3—C4	1.414 (4)
Ag—N	2.397 (2)	C3—H3A	0.9300
Ag—Ag^iii^	2.9200 (5)	C4—C5	1.416 (4)
O1—C10	1.252 (3)	C4—C9	1.415 (4)
O1—Ag^iv^	2.2067 (19)	C5—C6	1.413 (4)
O2—C10	1.252 (4)	C6—C7	1.366 (4)
O2—Ag^v^	2.252 (2)	C6—H6A	0.9300
N—C1	1.327 (4)	C7—C8	1.412 (4)
N—C5	1.374 (3)	C7—H7A	0.9300
C1—C2	1.395 (5)	C8—C9	1.375 (4)
C1—H1A	0.9300	C8—C10	1.517 (4)
C2—C3	1.367 (5)	C9—H9A	0.9300
			
O1^i^—Ag—O2^ii^	160.44 (9)	C5—C4—C9	119.7 (2)
O1^i^—Ag—N	109.04 (8)	C3—C4—C9	121.8 (3)
O2^ii^—Ag—N	90.51 (8)	N—C5—C4	121.6 (2)
O1^i^—Ag—Ag^iii^	84.15 (5)	N—C5—C6	119.7 (2)
O2^ii^—Ag—Ag^iii^	77.68 (6)	C4—C5—C6	118.8 (2)
N—Ag—Ag^iii^	156.92 (6)	C7—C6—C5	120.3 (2)
C10—O1—Ag^iv^	122.44 (18)	C7—C6—H6A	119.8
C10—O2—Ag^v^	128.3 (2)	C5—C6—H6A	119.8
C1—N—C5	117.8 (2)	C6—C7—C8	121.3 (2)
C1—N—Ag	112.45 (18)	C6—C7—H7A	119.3
C5—N—Ag	129.42 (18)	C8—C7—H7A	119.3
N—C1—C2	123.9 (3)	C9—C8—C7	119.4 (2)
N—C1—H1A	118.0	C9—C8—C10	119.2 (2)
C2—C1—H1A	118.0	C7—C8—C10	121.4 (2)
C3—C2—C1	119.5 (3)	C8—C9—C4	120.4 (3)
C3—C2—H2A	120.3	C8—C9—H9A	119.8
C1—C2—H2A	120.3	C4—C9—H9A	119.8
C2—C3—C4	118.8 (3)	O1—C10—O2	126.1 (3)
C2—C3—H3A	120.6	O1—C10—C8	117.1 (2)
C4—C3—H3A	120.6	O2—C10—C8	116.9 (2)
C5—C4—C3	118.4 (2)		

Symmetry codes: (i) −*x*+1/2, *y*+1/2, −*z*+1/2; (ii) *x*+1/2, −*y*+1/2, *z*−1/2; (iii) −*x*+1, −*y*+1, −*z*; (iv) −*x*+1/2, *y*−1/2, −*z*+1/2; (v) *x*−1/2, −*y*+1/2, *z*+1/2.
